# Using Machine Learning in Veterinary Medical Education: An Introduction for Veterinary Medicine Educators

**DOI:** 10.3390/vetsci10090537

**Published:** 2023-08-23

**Authors:** Sarah E. Hooper, Kent G. Hecker, Elpida Artemiou

**Affiliations:** 1 Department of Biomedical Sciences, Ross University School of Veterinary Medicine, P.O. Box 334, Basseterre KN0101, Saint Kitts and Nevis; 2 Faculty of Veterinary Medicine, University of Calgary, Calgary, AB T2N 4Z6, Canada; kghecker@ucalgary.ca; 3 International Council for Veterinary Assessment, Crystal Lake, IL 60014, USA; 4 School of Veterinary Medicine, Texas Tech University, 7671 Evans Drive, Amarillo, TX 79106, USA; elpida.artemiou@ttu.edu

**Keywords:** machine learning, veterinary medical education, random forest, medical education, artificial intelligence, Python, R, veterinary educators, educational data mining, learning analytics

## Abstract

**Simple Summary:**

Machine learning (ML) is subfield of artificial intelligence that enables computers to learn from data and improve their performance without being explicitly programmed by a human. ML has the potential to enhance veterinary medical education by improving learning, teaching, and assessments. This primer introduces ML concepts to veterinary educators and administrators, highlighting their similarities and differences with classical statistics. It then provides a step-by-step example using simulated veterinary student data to address a specific question: which records in the simulated veterinary student data will predict a student passing or failing a specific course. The example demonstrates the use of the Python programming language to create a random forest ML prediction model, a type of ML algorithm which is composed of many decision trees and each of these trees is composed of nodes and leaves. During the creation of the random forest model, we emphasize specific considerations such as managing student records which may have missing information. The results show how decisions made by veterinary educators during ML model creation may impact which type of records are shown to be most important. While this form of ML may prove to be beneficial, transparency in creating ML models is crucial, and further research is needed to establish best practices and guidelines for veterinary medical education ML projects.

**Abstract:**

Machine learning (ML) offers potential opportunities to enhance the learning, teaching, and assessments within veterinary medical education including but not limited to assisting with admissions processes as well as student progress evaluations. The purpose of this primer is to assist veterinary educators in appraising and potentially adopting these rapid upcoming advances in data science and technology. In the first section, we introduce ML concepts and highlight similarities/differences between ML and classical statistics. In the second section, we provide a step-by-step worked example using simulated veterinary student data to answer a hypothesis-driven question. Python syntax with explanations is provided within the text to create a random forest ML prediction model, a model composed of decision trees with each decision tree being composed of nodes and leaves. Within each step of the model creation, specific considerations such as how to manage incomplete student records are highlighted when applying ML algorithms within the veterinary education field. The results from the simulated data demonstrate how decisions by the veterinary educator during ML model creation may impact the most important features contributing to the model. These results highlight the need for the veterinary educator to be fully transparent during the creation of ML models and future research is needed to establish guidelines for handling data not missing at random in medical education, and preferred methods for model evaluation.

## 1. Introduction

In recent years, the field of health professions education has witnessed significant advances with the integration of machine learning (ML). ML, a subfield of artificial intelligence (AI), involves the development of algorithms and models that enable computers to learn from data and make predictions or decisions without being explicitly programmed. ML holds tremendous potential in creating adaptive learning platforms and intelligent tutoring systems, and has shown promise in the assessment and analysis of large-scale datasets including clinical records [1], diagnostic images [2], etc.

ML also offers the potential to enhance the learning, teaching, and assessments within veterinary medical education and may be incorporated across all aspects of veterinary medical education including admissions processes as well as student progress evaluations. Accordingly, veterinary educators must adapt to these rapid upcoming advances in technology and data science. As the field of veterinary medical education increasingly embraces data-driven approaches and evidence-based practices, understanding the fundamental differences and similarities between ML and classical statistics is paramount. Equally important is the recognition of the benefits, risks, and ethical dilemmas that may arise when utilizing machine learning within the veterinary education field. While a full discussion of machine learning ethics is outside the scope of this manuscript, veterinary educators should be aware of ongoing recommendations in the medical field to provide “machine learning literacy” to medical students and medical school faculty [3].

The purpose of this primer is to (i) introduce veterinary educators and veterinary college administrators to ML concepts, (ii) highlight similarities/differences between ML and classical statistics, and (iii) describe important considerations when using ML prediction models to answer hypothesis driven veterinary education questions.

This manuscript is divided into two sections. The first section defines educational data mining (EDM) and provides an overview of classical statistics commonly used in the veterinary medical education field. Information is presented alongside basic ML concepts and workflow to help illustrate the similarities and differences between these two main methodology categories. The second section provides a step-by-step worked example using simulated veterinary student data to answer a hypothesis driven question. Python syntax with explanations is provided within the text to create a random forest ML prediction model and within each step, specific considerations are highlighted when applying ML algorithms within the veterinary education field. A brief discussion follows highlighting additional considerations during the decision-making processes and interpretation of the random forest models after the initial models are constructed.

### 1.1. Introduction to Educational Data Mining and Machine Learning

Educational data mining (EDM) uses educational and student data and can potentially inform educational issues and learning environments [4,5]. Specifically, one of the key applications of ML within EDM is to better understand student progression in a degree program or course [5] and to develop prediction models to identify students at risk of not completing their degree program or a specific course [5,6]. EDM methodologies can be classified into three general methods, (1) classical statistical analysis (e.g., regression analysis), (2) artificial intelligence (e.g., neural computing), and (3) machine learning (e.g., random forests) [6]. This paper focuses on ML models and highlights uses of this methodology, although it is important to note that methodologies should be selected based upon the educational question that needs to be addressed.

The premise behind the use of ML is that it (1) enables computers to “learn” without requiring an individual to directly program it to do so [7,8] and (2) most often to build predictive models using big data or large, high-dimensional datasets [8]. A predictive model is a model which predicts future events or outcomes based upon the patterns found in the input data. The concept of big data can be viewed as large sized datasets (also known as large volume); datasets which contain diverse data such as numeric, text, graphics, etc. (also called wide variety); and allow quick generation of the data (also known as high velocity) [9]. For example, in a study assessing a medical school’s curricula, clustering ML techniques were employed to visualize the relationship between the learning objectives of courses and required competencies of medical students [10]. This type of question cannot be answered using classical statistical analysis. Statistical analysis may refer to the descriptive use, “to present and summarize data” [11] or the inferential use, “the process of drawing conclusions which have a wider applicability than solely to the sample of observations or measurements obtained”, and described in terms of probability or the likelihood of the occurrence of an event [11].

### 1.2. Comparison of Classical Statistical Analysis and Machine Learning Models

To help understand the difference between ML models and classical statistical analysis, assumptions, and the approach used to build such models or analyses, we discuss a recently published example of logistic regression that was used to evaluate if veterinary school admissions variable(s) would serve as predictors for students at risk of academic difficulty in the professional program. The statistical model building was structured following three main steps, (1) model specification, (2) parameter estimation, and (3) parameter probability distribution derivation [12].

During model specification, the authors calculated zero-order correlations [13], which means that the authors pre-determined potential correlations between two independent variables and only selected variables with zero-order correlations to include when building a single logistic regression model. The data included in the model adhered to specific assumptions including (1) the relationship between the logit (also known as log-odds) of the outcome and each of the included continuous independent variables were all linear, (2) there were no highly influential outlier data points, (3) there were no highly correlated independent variables (i.e., absence of multicollinearity), (4) the observations were independent of each other, and (5) the sample size was sufficient which is typically considered at least 10 observations of the least frequent outcome for each independent variable [14]. The parameter estimation was completed using a method such as maximum likelihood estimation (MLE), and subsequently tested the significance of each regression parameter based on the parameter distribution calculated previously [12].

While there are many different ML algorithms, the steps for constructing a model are quite similar and involve (1) model specification and (2) parameter estimation steps (i.e., training of the model). The approach to model specification and parameter estimation is different for ML algorithms as the model specification typically is data-driven rather than theory-driven [15]. This means that often, the parameter probability distribution derivation is not specified before training the ML model which results in ML models having better prediction power [12]. Furthermore, during the training of the ML model, many different empirical models need to be built using the ML algorithm. These initial models are built by the ML algorithm based on the relationships between the input and output variables using training sets of data. While the algorithm completes this step, the veterinary educator will need to make educated decisions when specifying the parameters for the final, best performing ML model. For example, the educator will need to specify certain parameters such as how many iterations should be performed for optimal performance of the trained model, and in response, the outputs of the ML models created from the training dataset will provide these answers. Furthermore, when constructing a ML model, it is important to note that each ML algorithm will have different assumptions or have no assumptions about the data. For example, with logistic regression models, five assumptions were listed above whereas many commonly used ML models do not make any assumptions about the data. Due to few to no assumptions about the data and because the training of the model typically consists of multiple datasets or resampling of the same dataset to form multiple datasets during parameter estimation within the ML model, highly correlated variables and outliers may be able to be included in the ML model. The same cannot be included in a logistic regression model [12,16,17] which could be disadvantageous for addressing some veterinary education and curricula questions.

In part 2 of this primer, we describe step by step, (i) how a training model is built, (ii) the different parameters of these models can be specified based upon the training of the model, and (iii) the specific assumptions for the algorithm selected in support of the working example.

### 1.3. Overview of the Main Types of Machine Learning Algorithms and Random Forest Machine Learning Models

Within ML, the algorithms employed are commonly categorized into four main categories, (1) supervised learning, (2) unsupervised learning, (3) semi-supervised learning, and (4) reinforcement learning [18]. In supervised ML, the veterinary educator serves as the “teacher” and the training data contain a range of predictors while the outcome is known. Following the veterinary school admissions example presented earlier, if each student was assigned a set of admissions variables and if it was known whether the student had academic difficulties (defined as dismissed from the DVM program or put on academic probation) or no academic difficulties, this could be used in the supervised model. In unsupervised ML, the outcome is unknown and instead the algorithm focuses on identifying relationships and groupings within the data [8]. In semi-supervised ML, the outcome is known for some of the dataset [18]. As such, utilizing the same veterinary school admissions example, the model would be trained only using student data with the known outcomes of academic difficulties or no academic difficulties. Immediately following, we would then iteratively apply the model to data with many unknown student academic difficulty outcomes. Reinforcement learning refers to the process when the machine/computer learns about its environment and chooses the optimal behavior to gain the greatest reward. The ML algorithm learns the behavior through trial and error, with some behaviors receiving rewards while other behaviors not deserving to receive rewards [18].

Selection of the ML algorithm is typically based upon the data structure type and the question being asked. In veterinary education, most data could be considered structured or unstructured. Briefly defined, structured data are typically stored in tabular format and follow a standard order such as student names, addresses, grades, etc. Unstructured data have no pre-defined format or organization, such as videos, audio files, presentations, and e-mails.

The example in this primer focuses on using structured data, which are simulated student records. We opted to use random forest as the example ML algorithm to help illustrate the steps for creating and evaluating a ML model. This model is one of the most utilized supervised learning algorithms and offers numerous benefits such as handling non-parametric data and being robust to outliers [6,19,20,21,22,23], both of which are commonly observed in veterinary educational data.

**Figure 1:**
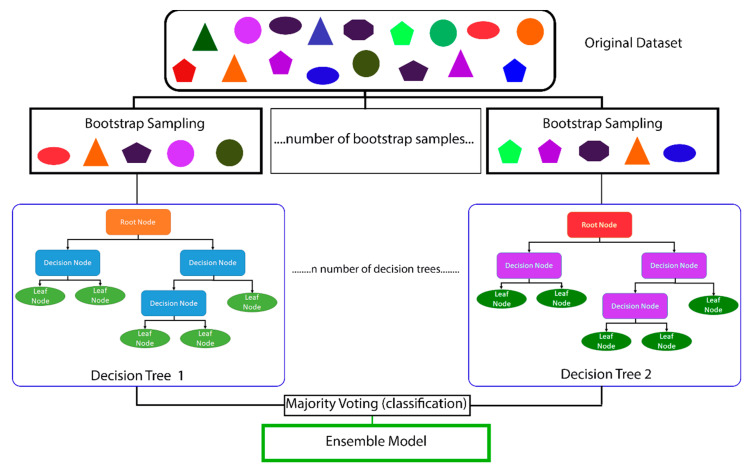
The original dataset is subset into many smaller, random datasets by the process known as bootstrap sampling. The number of bootstrap samples is equal to the number of decision trees that are created. Each decisions tree consists of a root node. If the data are true to the statement within the root node (e.g., a student’s Graduate Record Exam (GRE) > 300), all true data will go to the decision node on the left whereas all false data will go to the decision node on the right. The terminal nodes are known as leaf nodes. After the creation of each decision tree, the results of each decision tree are averaged (bagging) and the final prediction is the ensemble model as it is based upon the results of all the generated decision trees.

A random forest model is composed of decision trees as shown in Figure 1. Each decision tree is composed of nodes and leaves. The root node sits at the top of the decision tree and is the first division where the dataset is divided based upon whether the data are true or false. For example, if we asked whether a student practiced suture tying more than 15 h, if true, the data on the student move to the true decision node and if false, then they are assigned to the false decision node. At each subsequent node, the same division occurs with the student’s data being classified as “true” or “false” based upon that specific node’s statement (e.g., the student has more than 250 h of working as a surgical technician). The leaf node is the final output of the decision tree. Furthermore, Figure 1 shows how decision trees are a type of bagging (also known as bootstrap aggregating) ML algorithm. Bagging or bootstrapping is a method used to create smaller, random datasets out of the full dataset with replacement to estimate a population parameter [24].

Random forest models are a type of ensemble learning algorithm because the model contains many different decision trees which are then combined to produce the most effective optimal prediction model (Figure 1). In other words, rather than using a single hypothesis, an ensemble learning method will construct a set of hypotheses [25]—in our case, multiple decision trees. These hypotheses are assigned weights and voted upon by the ML algorithm which ultimately will result in providing the most important features contributing to the random forest models [20,22,25]. By creating many trees with a subset of the data, then combining the output of all trees, it helps to reduce over-fitting (i.e., the algorithm model trains the data too well and fails to be predictive for the testing data), reduces variance, and ultimately improves the model’s performance [20,22,25].

### 1.4. Programming Languages and Tools

**Figure 2:**
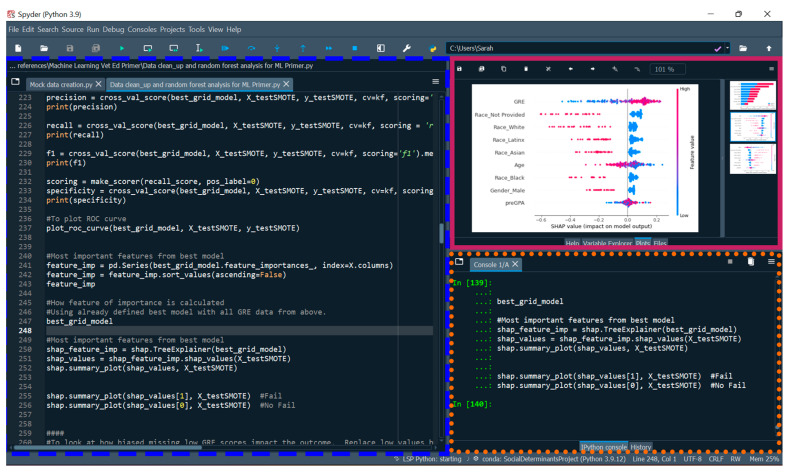
A screenshot showing the IDE Spyder with a few of the available panes or windows available. The Editor panel is outlined in a blue dashed line wherein the educator can create, open, and modify files with features such as autocompletion and syntax highlighting. The IPython console is outlined in an orange dashed line wherein the code is executed (Python code is run). The Plots window is outlined in a solid pink line and displays a beeswarm plot. Spyder is the IDE preferably utilized by the authors for Python projects and the IDE RStudio for R projects.

When constructing ML models, there are several different programming languages, tools, and software that can be used, each with different strengths. Here, we recommend veterinary educators use Anaconda Distribution, an open-source repository and toolkit. Anaconda is a platform that provides Python and R programming languages as well as a range of packages including a package management system [26]. An R or Python package is a collection of functions, compiled codes, sample data, and documentation in a well-defined format and is used to complete specific tasks or analysis. Within Anaconda, a designated environment is created specifically for a research study to avoid executing an installation or update that would disrupt packages or other frameworks such as integrated development environments (IDEs) (Figure 2). IDEs such as Spyder combine common developer tools with a single graphical user interface (GUI) and as such, not every action requires a line of code. Another way to think about Anaconda is to equate it to a mansion and a toolbox. Anaconda provides rooms where you can dedicate that room to a specific type of work or theme. Many times, the packages (i.e., the tools) will interact with each other or cause problems, which is why it is important to dedicate a specific “room” in the mansion for each project you are working on, as then, once your model is complete, it will always run within that room without issues. If you update or install a new package for a different project, it will not affect the other projects. This highlights that it is essential to report the version of the IDE, the programming language and the package version, used in creation of the ML models as not all versions may be compatible [27].

## 2. Simulation of Dataset and Creation of a Random Forest Machine Learning Model

**Figure 3:**
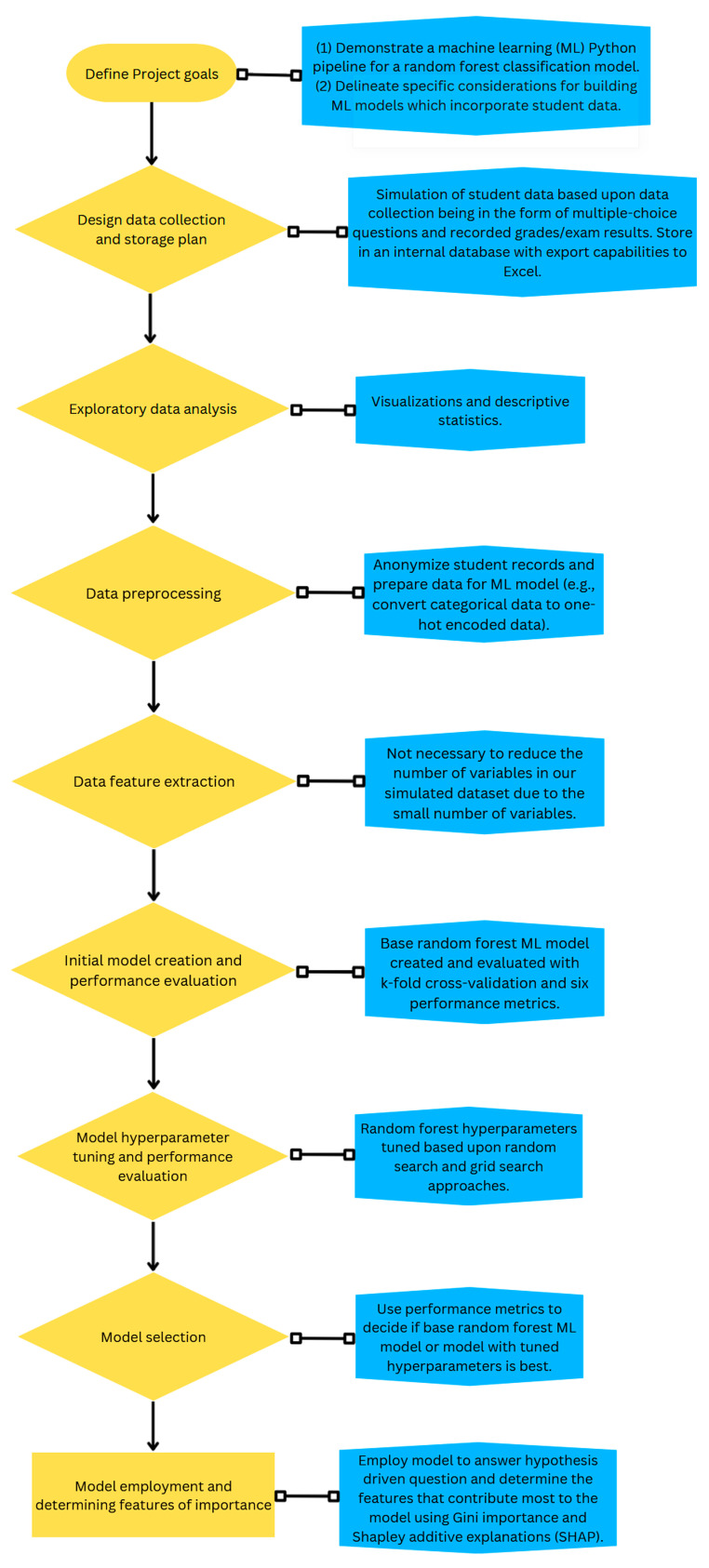
A flowchart highlighting the key steps for a machine learning project which are detailed in this primer is shown in orange. In blue describes the specific outcomes or processes that we will describe in our working example.

In this second part of the primer, we provide a working example of creating a ML model using simulated data and Python programming language to answer a hypothesis driven research question. Figure 3 shows a visual workflow of the working example. Within each step of the process, important considerations for the veterinary educator community will be discussed and it will be illustrated how data quality and decisions by the educator may impact the ML model.

### 2.1. Defining the Project Goals

The first step requires defining the problem or hypothesis and determining the data needed to answer the question. Here, we explore two project goals. The first project goal is to demonstrate a ML Python pipeline for creating a random forest classification model from simulated data. The purpose of this ML model is to identify the most important predictors utilizing a simulated dataset that contributes to students failing a course during the pre-clinical years of veterinary school training. Our hypothesis suggests that students’ GRE scores are the most important predictor for determining if a student will pass or fail a course in the Doctor of Veterinary Medicine (DVM) program. Our second project goal delineates specific considerations for building ML models which incorporate veterinary student data.

### 2.2. Data Collection and Storage Plan

After defining the project goals, it is critical to design a data collection and storage plan. The quality and performance of the ML models depends on the quality of data that are collected [28]. A good data collection strategy and well-designed storage medium for the dataset is essential for appropriate analyses and interpretation (i.e., database or Excel spreadsheets). For this to occur, the veterinary educator should have a basic understanding about the model being used. This requires educators to determine if the raw data can be used in the model or if different transformation processes will need to be used first. For example, a random forest model cannot handle non-numeric data; therefore, if a student’s demographic data are collected, they will need to be transformed (transformations are discussed in Section 2.4). When collecting the demographic data, it is important to decide if it should be collected by multiple choice options or free-text entry. Multiple choice may limit responses if a student does not identify with the options, but if free-text entry is used, then the veterinary educator will need to ensure that all answers entered are identical in format and spelling (i.e., the programming languages will view “black” and “Black” as two different ethnicities) and have pre-defined groups (e.g., Will Hispanic and Latinx be grouped together or separately?). Additionally, some random forest models cannot handle missing data, and so the veterinary educator should establish a plan for how to deal with an incomplete student record. By addressing these considerations, we can prevent a variety of problems that arise from poorly defined or poorly collected datasets such as ensuring the data collected are adequate to answer the hypothesis or question [12,29].

#### 2.2.1. Simulated Data Collection and Storage

The dataset format is based upon OutReach IQ [30], an internal database of students enrolled at Ross University School of Veterinary Medicine (RUSVM) where all data can only be viewed by faculty with approved Institutional Review Board (IRB) protocols or IRB exemptions to maintain student confidentiality. For this example, three simulated datasets were created, and each contains 400 simulated student records. To keep the analysis simple, the number of variables in the simulated datasets are limited to: Full name, gender, ethnicity/race, age, pre-admission GPA, and GRE (Table 1). All three datasets are imbalanced, meaning those passing and failing a course are not equal. All three datasets have 10% of the students failing a course and 90% not failing a course. All three datasets have identical values except for the GRE value as this will represent commonly missing GRE values, a result of not being an admission requirement for many veterinary schools. In the dataset named “BiasedGRE1”, we removed the lowest GRE from 14 of the students who experienced failure and from 71 of the students who did not experience failure. In the dataset named “BiasedGRE2”, we randomly removed GRE scores from 200 student records. The simulated datasets and all code for the models created in this manuscript are available at https://github.com/RUSVMCenter4/Veterinary_Education_ML_Tutorial (accessed on 14 May 2023) [31].

#### 2.2.2. Importing the Dataset

The first step to creating a ML model is to load the required Python packages and the dataset(s). We used Python version 3.11.1 within Spyder version 5.1.5 for all coding. To load our datasets, we used a package called pandas [32], version 1.4.3. Any characters or text after a pound sign (#) are not read by Python and this provides a way to insert notes into the Python code.

#Import required packages:
Import pandas as pd
#Import the dataset using the function pd.read_excel().
Dataset = pd.read_excel(r’C:\location_of_data\name_of_excell_datafile.xlsx’,
sheet_name = ‘name’)
#To view the first 10 rows of the dataset with the column names:
Dataset.head(10)

### 2.3. Exploratory Data Analysis

Once the dataset is loaded, we recommend that data visualization and descriptive statistics are completed prior to moving to the next step. This helps the veterinary educator to understand if the data contain outliers, missing data, the distribution of variables, and much more. We are limited in further expanding upon this critical step considering that we randomly generated our small dataset and selected the distribution of the values.

### 2.4. Data Preprocessing

Data preprocessing describes the process of when the raw data are prepared for training and testing the ML model. As a first step in protecting student confidentiality, we recommend that student records be assigned a randomized ID, and the list of names along with the assigned random IDs be stored safely per IRB standards at your institution. This can be easily done in Python using a for loop with a random number generator function from the random module which is built-in to Python. A for loop in Python is a line of code which repeatedly uses, or iterates, a function, and in this case, we repeat the function 400 times. This is equal to the number of student records in our dataset. All python code for this step is available at https://github.com/RUSVMCenter4/Veterinary_Education_ML_Tutorial (accessed on 14 May 2023) and shows how to generate random student IDs and then add the IDs as a column to the dataset [31]. This step is not included here because we simulated the entire datasets. Additionally, in this manuscript, we do not address all steps and considerations when making a dataset completely anonymous, and therefore we recommend an expert be consulted if outcomes of the ML project are made public.

**Figure 4:**
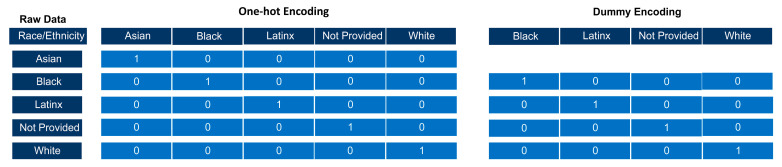
The race/ethnicity raw data is composed of categorical variables which are converted to numerical values using one-hot encoding. There are now five columns for race with one-hot encoding as one-hot encoding adds a new binary column for each category. Students who identify as Asian will have a 1 in the Asian column and a 0 in all other columns.

We use the random forest algorithm provided in the Python package scikit-learn [33]. To begin to prepare the datasets for this ML algorithm, categorical data such as gender and ethnicity/race must be converted to numerical values. This is accomplished through one-hot encoding or dummy encoding. One-hot encoding ensures a rank is not assigned to categorical variables while the variable is converted into numerical data. One-hot encoding adds a new binary variable for each unique categorical value and the original encoded variable is removed (Figure 4). Dummy encoding uses binary variables and creates the number of columns equal to the number of categories minus 1 (Figure 4).

#To one-hot encode for race column, use the get_dummies() from the pandas package
#We assign this transformed data to a new variable called dataset_OneHot
#We also need the argument drop_first to not be true in order to perform one-hot
#encoding.
Dataset_OneHot = pd.get_dummies(dataset, columns = [“Race”], drop_first = False)
dataset_OneHot.head() #To view the first several rows and column names

It is important to note that Python functions have different arguments or parameters, and when these arguments have an assigned value, they are used when the function is performed. To perform dummy encoding (Figure 4, we will need to set the argument drop_first to true.

#To dummy encode the gender column
dataset_OneHot = pd.get_dummies(dataset_OneHot, columns = [“Gender”],
drop_first = True)
print(dataset_OneHot.head()) #To view the first several rows and column names

For continuous variables such as age, pre-admission GPA, and GRE, scaling the variables, commonly known as feature scaling, or standardizing the variables may need to occur. When employing feature scaling techniques, our goal is to make sure that all the variables are on the same scale or nearly the same scale. This will not change the distribution of the data. This means this step will not transform non-parametric data into normally distributed data. For example, within our dataset, age ranges from 20 to 40 whereas GPA ranges from 3.00 to 4.00 and GRE ranges from 260 to 330. If we were using a ML model that was unsupervised and cluster based upon relationships and groupings [8], leaving these values unscaled could impact the results due to many models being based upon Euclidean Distance, or the distance between two data points.

We kept age as a continuous variable as random forests are not a distance-based classifier, robust to outliers, and do not need parametric data [23]. We also kept pre-admission GPA and GRE scores continuous because random forests handle high non-linearity between independent variables [34]. Non-linear parameters typically do not affect the performance of the decision tree models because the splitting of the decision nodes is based upon absolute values, “yes” or “no” (Figure 4), and the branches are not based upon a numerical value of the feature [20,21,23,34,35].

GRE is a continuous variable, with two of the simulated datasets containing missing GRE scores. It is important to determine if the data are missing not at random (MNAR) due to a specific reason (i.e., a student does poorly on the GRE and so does not report the result), or if the missing data are missing at random (MAR) or missing completely at random (MCAR). MAR data are missing and while randomly missing, can be explained by another observed variable (i.e., a dataset contains information on medical absences and course exam grades, a student with a missing course exam grade could be explained if they had a medical absence). MCAR data refer to missing data that are randomly distributed across the variable and is not related to the other variables (i.e., if the dataset contained a few student records without GPA scores due to human error inputting the scores into the student record database). GRE was chosen as an example variable for missing data because the GRE is recommended or no longer required for many veterinary professional programs as there are concerns the GRE may hinder diversity and inclusion efforts and may be a burden for low-income students [36,37]. How the veterinary educator decides to handle these data will impact the results of the ML model. This will be shown when reviewing the results of the ML models created using the three datasets.

Unfortunately, currently there are no established methods for handling MNAR data in medical education, and therefore there are no guidelines on imputation methods, or the action of replacing missing values in the dataset with an alternative or predicted value. Our code below demonstrates loading 1 of the 2 biased GRE datasets and use listwise deletion (deletion of the student recording containing NAs) and substitution (the mean or median value for the column) [38,39], which are two common methods reported in education and often are the default methods in R and Python packages.

#Import the first dataset with missing GRE values using the function pd.read_excel().
Biased_dataset = pd.read_excel(r’C:\location_of_data/name_of_excell_datafile.xlsx’,
sheet_name = ‘name’)
#Code to drop delete each student record that does not have a GRE score reported
#The “empty” GRE value will be noted as an “na” in Python, therefore we use the
#dropna()
#The argument axis = 0 means the row with the “na” will be dropped.
#The argument how = ’any’ means that any “na” will result in the row being deleted
#The argument inplace = True means that a new dataframe will not be created
biased_dataset_OneHot.dropna(axis = 0, how = ‘any’, inplace = True)
#Code to replace each missing GRE score with the mean of the GRE value
biased_dataset_OneHot.fillna((biased_dataset_OneHot[‘GRE’].mean()), inplace = True)

The full code to create the base random forest models using the two missing GRE datasets is available on Github [31]. These datasets with missing GRE were created to illustrate NMAR and MCAR data and how commonly accepted methods for handling these data types in the literature have the potential to affect the model; therefore, only base random forest models were created and no hyperparameter search was conducted.

### 2.5. Data Feature Extraction

Feature extraction, also commonly referred to as feature selection or dimension reduction, refers to when different techniques are used such as principal component analysis (PCA) or stepwise regression to reduce the number of variables into the model [40]. Our original datasets contained 5 variables after removing the student names. After one-hot encoding, our datasets expanded to 9 variables. These datasets are quite small and do not need to undergo dimension reduction or reducing the number of variables (i.e., feature selection). However, veterinary educators need to be aware that having datasets with high dimensionality will require higher computational power to run the ML algorithm [41]. More importantly, some ML algorithms may have lower predictive performance and have the potential to fail to provide meaningful results when there are a large number of variables [41,42].

### 2.6. Model Creation and Performance Evaluation

ML algorithms learn from the input dataset which is typically divided into training and testing datasets. The training dataset is used to train the ML model followed by evaluating the model with the testing dataset. Splitting the dataset is commonly done to help reduce the risk of over-fitting. If over-fitting occurs, this means the model only performs well on the data used to train it, and the model’s performance is reduced when it is applied on new data [43]. We recommend that multiple models be trained with different parameters and compared to find the best candidate model for the identified educational research question. This is commonly done by what is termed as a ML pipeline. The parameters within the pipeline can be adjusted in any of the steps, (1) data pre-processing, (2) feature extraction, (3) model training, and (4) model evaluation. Model evaluation is completed by comparing a variety of different metrics which may include accuracy, F-scores, receiver operating characteristic (ROC) curves, and others which we will expand upon more in the experimental section.

#### 2.6.1. Generation of Base Random Forest Model

The first step of creating a ML model, including random forests, is to take the dataset and separate the variables into one dataframe (a table with rows and columns) and the target column in a second dataframe. The target column is the outcome we wish to predict. Within our example, we are creating a classification model, so the outcome is a binary outcome, either a “Pass” or a “Fail. In the “Fail” target column, a 0 indicates a student did not fail a course and 1 indicates a student failed a course.

#X is our variable dataframe and y is our target dataframe
#create dataframe without target, [rows, columns], the : indicates to select all rows
X = dataset_OneHot.loc[: , dataset_OneHot.columns != ‘Fail’]
y = dataset_OneHot[‘Fail’] #target variable for prediction

We are most interested in determining what variables lead to the outcome or target column. Considering that the simulated dataset is composed of a majority and minority class (target variable does not have an equal number of 0 s and 1 s), the dataset has an imbalance bias, one of the three main types of recognized biases in ML [44]. The two main approaches to deal with imbalanced target variables is to use an oversampling technique which creates additional minority classes (student records with a course failure) or undersampling techniques to randomly delete majority classes (student records without a course failure). We selected to turn our dataset into a balanced dataset by using an oversampling method called synthetic minority oversampling technique (SMOTE) which is a common method for dealing with models predicting student success in higher education [45,46,47]. After performing SMOTE, our dataset will result in students who failed a course being equal to the number of students who did not fail a course.

#Import required python function of SMOTE from Python package imbalanced-learn
#version 0.10.1from imblearn.over_sampling import SMOTE,
#Oversampling to allow 0 and 1 target to be equal
#Assigning a value to the random state argument ensures that anyone can generate
#the same set of random numbers again
X_resampled, y_resampled = SMOTE(random_state = 23).fit_resample(X, y)

With a balanced target variable, we are now ready to split our dataset into training data and testing data. Splitting the dataset is commonly done to help reduce the risk of over-fitting. As our code shows below, we used train_test_split() from scikit learn Python package (version 1.1.1) to split our dataset. We also provide code showing the data structure as the length dataframes containing the variables must be the same length as its counterpart containing the target, otherwise the random forest model will not be constructed.

#Import required functions:
from sklearn.model_selection import train_test_split
#Use the balanced data to create testing and training datasets with 70% of the data
#being training and 30% of the data being testing.
X_trainSMOTE, X_testSMOTE, y_trainSMOTE, y_testSMOTE =
train_test_split(X_resampled, y_resampled, stratify = y_resampled, test_size = 0.3,
random_state = 50)
#Check sizes of arrays to make sure it they match each other
print(‘Training Variables Shape:’, X_trainSMOTE.shape)
print(‘Training Target Shape:’, y_trainSMOTE.shape)
print(‘Testing Variables Shape:’, X_testSMOTE.shape)
print(‘Testing Target Shape:’, y_testSMOTE.shape)

Once we have our training and testing dataset, we are ready to construct the base random forest model with the arguments, or parameters, being left at their default values. The model must first be built and then trained using the training data.

#Import required functions:
from sklearn.ensemble import RandomForestClassifier
#Build base model without any changes to default settings
forest_base = RandomForestClassifier(random_state = 23)
#Train the model via fit()
forest_base.fit(X_trainSMOTE, y_trainSMOTE) #using training data

#### 2.6.2. Evaluation of the Base Random Forest Model

Once the base ML model is created, then we evaluate how well the model performs when it is shown new data, the test data. ML model performance can be assessed using different techniques which are discussed individually below. Each of the model performance methods uses a range from zero to one, with one indicating perfect performance and zero indicating all predictions were wrong. We recommend that cross-validation (CV) be included in each of the performance metric calculations.

**Figure 5:**
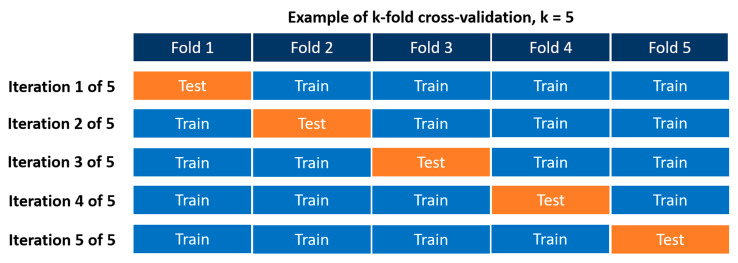
An example of a k-fold cross validation with k = 5. Five iterations of the training dataset are evenly divided into five folds, four of which are used for training and the last one for testing the model. This is repeated five times. The final validation would take place using the testing dataset (not shown).

CV is used to help detect overfitting while evaluating the performance of the ML model on new data. We used k-fold cross validation, which is one of the most common cross validation techniques [48]. In k-fold cross validation, the dataset is divided into folds (consider these as subset datasets) based upon the assigned k-value. One fold is saved as a validation dataset and the other folds are used to train the model. As Figure 5 shows, this process is repeated multiple times with each repeat holding out a different fold for validation of the model. The results from each validation set are averaged to produce the final performance value. Typical k-values are 3, 5, and 10, but there are no established rules guiding the selection of these values.

To prepare our model for the performance metrics, we must define the k-value for the CV and input our testing dataset into the trained random forest model. The trained random forest ML model will predict the target category or outcome of whether a student failed a course or not based upon what it learned from the training dataset.

#Make predictions using testing data set
y_predictions = forest_base.predict(X_testSMOTE)
y_trueSMOTE = y_testSMOTE #Rename the test target dataframe
#Import required function
from sklearn.model_selection import KFold
#Defining the cross-validation to be able to compute the performance metrics using
#the k-fold CV
kf = KFold(shuffle = True, n_splits = 5)

Overall accuracy, recall (i.e., sensitivity or true positive rate), specificity (i.e., true negative rate), precision, F-score (i.e., F1-score), and receiver operating characteristic (ROC) curves are performance metrics which are computed using the true negative (TN), false negative (FN), false positive (FP), and true positive (TP) values. A confusion matrix summarizes the results of the random forest classification algorithm and defines these values as shown in Table 2.

The *overall accuracy* is determined by the proportion of the total number of student predictions that were correct over all type of predictions made, and can be calculated using Equation (1):

(1) $$Overall\;accuracy=\frac{\left(TN+TP\right)}{\left(TP+TN+FP+FN\right)}$$

#Import required function
from sklearn.model_selection import cross_val_score
#To calculate the accuracy of the model using k-fold cross validation
score_accuracy_mean = cross_val_score(forest_base, X_testSMOTE, y_trueSMOTE,
cv = kf, scoring = ‘accuracy’).mean()
print(score_accuracy_mean) #View the mean of the CV validation results for
#accuracy of the model.

The true positive rate (*TPR*), also known as sensitivity or recall, is defined as the proportion of students who failed a course which were correctly classified as having failed a course and was calculated using Equation (2):

(2) $$recall\;or\;sensitivity\;or\;TPR=\frac{TP}{\left(TP+FN\right)}$$

#To calculate the recall of the model using k-fold cross validation
recall = cross_val_score(best_grid_model, X_testSMOTE, y_testSMOTE, cv = kf,
scoring = ‘recall’).mean()
print(recall) #View the mean of the CV validation results for recall of the model

The true negative rate (*TNR*), also known as specificity, is defined as the proportion of students who did not fail a course which were classified correctly as not failing and was calculated using Equation (3):

(3) $$specificity\;or\;TNR=\frac{TN}{\left(TN+FP\right)}$$

There is no *specificity or TNR* option in the cross_val_score() and so we must define specificity using the make_scorer() function.

#Import required function
from sklearn.metrics import make_scorer
#Define specificity
scoring = make_scorer(recall_score, pos_label = 0)
#Use our defined specificity as the type of score that is calculated
score_specificity_mean = cross_val_score(forest_base, X_testSMOTE, y_trueSMOTE,
cv = kf, scoring = scoring).mean()
cross_val_score(forest_base, X_testSMOTE, y_trueSMOTE, cv = kf, scoring = scoring)
print(score_specificity_mean) #View the mean of the CV validation results for
#specificity of the model

*Precision* is the number of correct predictions a student failure out of all students that were classified as experiencing a failure and was calculated using Equation (4):

(4) $$Precision=\frac{TP}{\left(TP+FP\right)}$$

# To calculate the precision of the model using k-fold cross validation
score_precision_mean = cross_val_score(forest_base, X_testSMOTE, y_trueSMOTE,
cv = kf, scoring = ‘precision’).mean()
print(score_precision_mean) #View the mean of the CV validation results for
#precision of the model

The *F-score* (*F*1) is a weighted harmonic average of precision and recall and is calculated using Equation (5):

(5) $$F\text{-}score\;or\;F1=2\times \frac{\left(precision\times recall\right)}{\left(precision+recall\right)}$$

#To calculate the *F-score* of the model using k-fold cross validation
score_f1_mean = cross_val_score(forest_base, X_testSMOTE, y_trueSMOTE, cv = kf,
scoring = ‘f1’).mean()
print(score_f1_mean) #View the mean of the CV validation results for precision of
#the model

Receiver operating characteristic (ROC) curves are created by plotting the sensitivity versus the specificity at different cut points for binary classification models [49]. The area under the ROC curve (AUC) is calculated from the ROC curves and is the last validation method we will use to assess each model. This single numerical score is considered a superior method compared to accuracy when evaluating the performance of prediction models [50].

#To calculate the ROC curve AUC of the model using k-fold cross validation
#score_auc_mean = cross_val_score(forest_base, X_testSMOTE, y_trueSMOTE, cv =
kf, scoring = ‘roc_auc’).mean()
print(score_auc_mean) ) #View the mean of the CV validation results for ROC curve
#AUC of the model

#### 2.6.3. Tuning of the Random Forest Model

Now that we know our model results, we can try to use data-driven approaches to improve the performance of our model. This means we will try to tune our model’s hyperparameters to improve the performance of the model. First, we will define a list of hyperparameters, or the parameters that are specific before training the model. This will help determine the best parameters that are learned during the training process of the model. We will demonstrate two common data-driven hyperparameter approaches, (1) Random search [51] and (2) Grid Search [52]. We first use random search as it requires lower computational power and will test a user-specified random number of combinations in the hyperparameter grid. Once we have the best estimates using random search, we will define a new hyperparameter grid with values closer to the selected output values from the random search. We will then use grid search, which will look at every possible combination in the hyperparameter grid.

##Assess hyperparameters to try to improve upon base model:
#Import required functions:
from sklearn.model_selection import RandomizedSearchCV
from sklearn.model_selection import GridSearchCV
# Create the hyperparameter grid for first the random search function
hyper_grid = {# Number of trees to be included in random forest
     ‘n_estimators’: [150, 200, 250, 300, 350, 400],
# Number of features to consider at every split
     ‘max_features’: [‘sqrt’],
#Maximum number of levels in a tree
     ‘max_depth’: [10, 20, 40, 60, 80, 100, 120, 140, 160, 180, 200],
# Minimum number of samples required to split a node
     ‘min_samples_split’: [2, 4, 6, 8, 10],
# Minimum number of samples required at each leaf node
     ‘min_samples_leaf’: [1, 2, 4, 6, 8, 10],
# Method of selecting samples for training each tree
     ‘bootstrap’: [True, False]}
#Initiate random forest base model to tune
best_params = RandomForestClassifier(random_state = (23))
#Use random grid search to find best hyperparameters, uses k-fold validation as cross
#validation method
#Search 200 different combinations
best_params_results = RandomizedSearchCV(estimator = best_params,
param_distributions = hyper_grid, n_iter = 200, cv = kf, verbose = 5, random_state = (23))
#Fit the random search model
best_params_results.fit(X_trainSMOTE, y_trainSMOTE)
#Find the best parameters from the grid search results
Print(best_params_results.best_params_)
#Build another hyperparameter grid using narrowed down parameter guidelines
#from above
#Then use GridSearchCV method to search every combination of grid
new_grid = {‘n_estimators’: [250, 275, 300, 325, 332, 350, 375],
       ‘max_features’: [‘sqrt’],
       ‘max_depth’: [160, 165, 170, 175, 180, 185, 190, 195],
       ‘min_samples_split’: [1, 2, 3, 4, 5, 6],
       ‘min_samples_leaf’: [1, 2, 3],
       ‘bootstrap’: [True]}
#Initiate random forest base model to tune
best_params = RandomForestClassifier(random_state = (23))
#Use GridSearchCV method to search every combination of grid
best_params_grid_search = GridSearchCV(estimator = best_params, param_grid =
new_grid, cv = kf, n_jobs = −1, verbose = 10)
#Fit the gridsearch model
best_params_grid_search.fit(X_trainSMOTE, y_trainSMOTE)
#Get the results of the search grid form the random forest model
best_params_grid_search.best_params_
#Using the results of the best parameters, we will create a new model and show the
#specific arguments.
best_grid_model = RandomForestClassifier(n_estimators = 375, max_features = ‘sqrt’,
max_depth = (160), min_samples_split = 2, min_samples_leaf = 2, bootstrap = True)
#Best model based upon grid
best_grid_model.fit(X_trainSMOTE, y_trainSMOTE)

After creation of the model, the performance metrics can be calculated, and the veterinary educator will need to decide which model is best, the base model or the model using the new parameters.

#### 2.6.4. Determining the Most Important Features of the Random Forest Model

After selecting the best model based upon the performance metrics, we need to determine which features contribute most to the model being able to predict the outcome of a student. Each feature has a score. A higher score means it contributes more to the model’s prediction whereas a lower score indicates the feature has a lower contribution to the model’s prediction. There are a variety of approaches to calculating the feature importance values, and some approaches depend on the ML algorithm selected whereas others can be used for a variety of ML algorithms [12].

We will use two methods to assess the features of importance, the Gini importance (or mean decrease Gini) and the visual Shapley additive explanations (SHAP) (Shap python package version 0.40.0). Gini importance is the most common method used to determine the relative depth or rank of a feature used as a decision node within the random forest model [20,53]. The most important features have a larger value and will be located most often at decision nodes near the top of the individual trees (Figure 1). By being near the top of the tree, a larger percentage of the input samples are utilized by that specific decision node. This means that the feature contributes more to the final prediction decision compared to decision nodes lower on the tree [33,53]. Features of importance determined by SHAP are based upon classic game-theoretic Shapley values. SHAP measures local feature interaction effects and helps provide a better understanding of the overall model [54] based on combining the explanations for each student outcome that is predicted.

#Most important features from best performing random forest model, Gini im
#portance
feature_imp = pd.Series(best_grid_model.feature_importances_, index = X.columns)
feature_imp = feature_imp.sort_values(ascending = False)
print(feature_imp)
#Import required package
import shap
#Most important features from best performing random forest model, SHAP values
shap_feature_imp = shap.TreeExplainer(best_grid_model)
shap_values = shap_feature_imp.shap_values(X_testSMOTE)
shap.summary_plot(shap_values, X_testSMOTE) #Shows results in a plot

Model employment:

The best performing model is selected and used to address the educational research question or problem.

## 3. Results

In the methods, we describe how a total of five random forest models were created. The full code provided resulted in the production of two random forest models, a model using the default parameters and a model with parameters selected after hyperparameter tuning. Table 3 shows the performance metrics for both models which were built from the complete dataset without any missing values.

The most important features, as ranked by the Gini criterion, varied depending on which dataset was used to train and test the model as well as the imputation or replacement method selected to address the missing values. The full list of most important features from each model are in Table 4.

**Figure 6:**
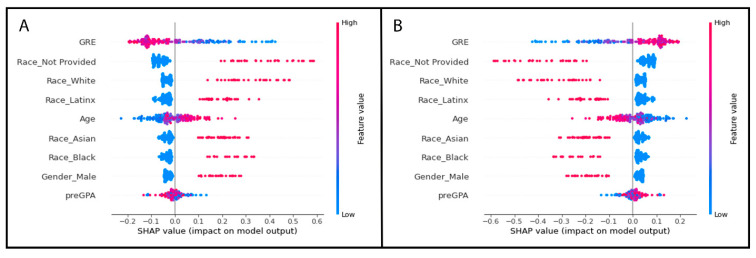
Beeswarm plots of the simulated dataset containing no missing GRE values. Each dot represents a student record with the feature value represented by a color. As the feature value color bar shows, a higher value is pink and a lower value is blue. The *x*-axis shows the SHAP values for the most important features listed along the *y*-axis. The *y*-axis values represent the features of importance with the top variable being the most important and the subsequent ones organized in descending rank of importance. (**A**) shows the SHAP values for students who did not fail any course, (**B**) shows the SHAP values for students who failed a course.

**Figure 7:**
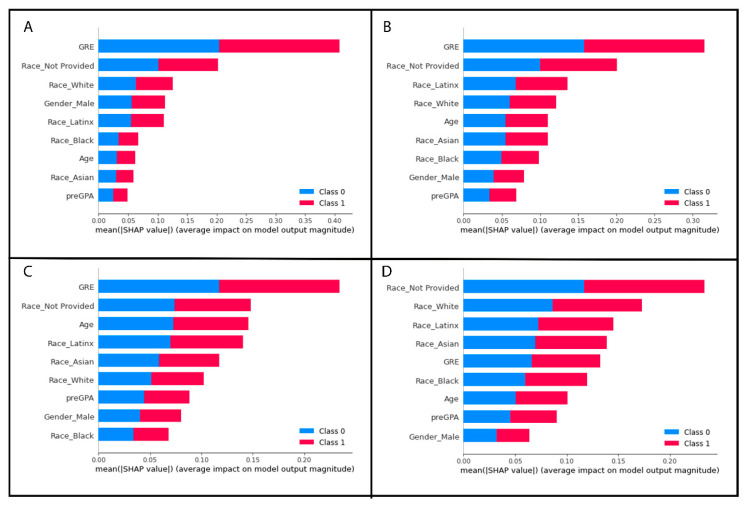
Global feature importance plots based on SHAP values for the datasets with missing GRE values. The *y*-axis values represent the features of importance with the top variable being the most important and the subsequent ones organized in descending rank of importance. The mean absolute value for each feature for all student records in the dataset is displayed on the *x*-axis. (**A**) Shows the features of importance for the simulated data where student records were eliminated with missing low GRE values. (**B**) Shows the features of importance for the simulated data where student records with missing low GRE scores were replaced with the mean GRE score of all applicants. (**C**) Shows the features of importance for the simulated data where student records were eliminated with random missing GRE values. (**D**) Shows the features of importance for the simulated data where student records with randomly missing GRE scores were replaced with the mean GRE score of all applicants.

GRE and three of the one-hot encoded categorical variable levels from the race/ethnicity column were identified by SHAP values as the most important features of the best-performing random forest model without any missing values. The results are plotted as summary beeswarm plots in Figure 6. Figure 7 shows the SHAP-determined most important features of the four random forest models with missing GRE values and are visually displayed as summary bar plots ranking the most important features at the top of the *y*-axis.

## 4. Discussion

ML has the potential to become a powerful tool in veterinary education; however, ML algorithms have yet to be tapped by veterinary educators. This manuscript focuses on making ML more accessible by providing a practical overview of ML and the creation of a supervised ML algorithm, a random forest ML model. As an emerging tool, there are some important considerations that veterinary educators must be aware of when employing ML models. We further highlight some of these considerations below which we initially present in the working example section when creating the models by discussing the results of the different models created by the different datasets.

Two of the most important considerations are the quality of a dataset and when it contains incomplete records. As previously discussed, missing data can be categorized into three main types. In education studies, often, the missing data will be MAR (e.g., a student who is sick misses an exam and can be explained by a leave of absence) or NMAR (e.g., a student chooses to not participate in an active learning exercise or chooses to not complete a teaching evaluation). NMAR data are non-ignorable data, meaning simply eliminating these students from the analysis will result in bias. In the literature, there are well accepted methods for handling MCAR or MAR data in education [6,38,39,55,56], but there are not commonly accepted methods for NMAR data in medical or veterinary education fields.

We utilized two pre-replacing methods that have been reported to be two of the most frequently encountered techniques in the higher education literature [6,57], listwise deletion and imputation (i.e., replacement). While there are many different imputation methods, we selected a single imputation method over multiple imputation (the creation of many datasets with the model results averaged). Our decision in part was to help maintain the simplicity of our model for this introductory manuscript and to emphasize to the reader the importance of recognizing and handling NMAR and MAR data. In Table 4, our random forest model without missing data revealed that GRE, age, and race/ethnicity not reported were the top three features that contributed most to our prediction model when Gini criteria were used. When approximately 20% of the lower GRE values were eliminated, listwise deletion resulted in GRE contributing more to the model with race/ethnicity not provided and pre-vet school GPA being the second and third most important feature. When we used single imputation with the mean, our top three most important features were GRE, pre-vet school GPA, and age. Keeping in mind that our dataset has very few variables, and on a dataset with more features, it is possible to see even more of a dramatic effect. The two random forest models created with the MAR or MCAR GRE dataset shows listwise deletion and single imputation were more similar than the NMAR GRE dataset. However, our simulation shows that both listwise deletion and replacement with the mean still impacted the random forest model with only the top two of the three top contributing variables being the same. This supports that in the education field, there is a large gap of knowledge in how to deal with missing data within student records and should be an area of future research. Until this can be addressed, we recommend that any veterinary education studies choosing to use ML clearly state why they selected the reported method for handling missing data, and to consider using multiple replacement methods to compare the results between models and provide a better understanding of the impacts of each replacement method.

Another important consideration that must be made when constructing ML models is to decide how the decision trees are constructed and how the features of importance will be determined. We opted to use the Gini index as the impurity function within our code. This is the default option in the random forest function within the scikit learn package [33]; however, in random forest models, permutation importance is also commonly used [20]. While a full discussion between these methods is beyond the scope of this manuscript, we selected the most commonly reported method in the EDM literature and, if we accept a greatly oversimplified explanation, the Gini index (or Gini impurity criterion) can be considered a robust and reliable impurity function and in part, it has been attributed to helping to reduce the errors in prediction when combining the individual decision trees into the random forest [16,20]. It is important to recognize that the Gini impurity criterion is not perfect, and in our veterinary education datasets where there is a mix of continuous variables, binary variables, and categorial variables, the Gini impurity criterion tends to overestimate the importance of continuous variables [20].

We can see how the continuous variables of GRE, age, and pre-vet GPA are in the top four most important features based upon the Gini criterion (Table 4), but this changes when we evaluate our model using the SHAP method to determine the features of most importance. The SHAP method for estimating the features of importance in decision trees, which uses the game-theoretic Shapley values as a basis, was proposed in 2020 [54], and is beginning to gain popularity due to the SHAP method results relying heavily on a very visual based report. The visual aspects of the SHAP method help to communicate the models to non-technical stakeholders and provide a way for us to view the contribution of each variable in the model for each row of student data (Figure 6).

The bar plots (Figure 7) show the mean absolute SHAP value with the most important feature having the largest value and being at the top of the plot. As the SHAP value, or feature importance, decreases, it descends along the *y*-axis. The beeswarm plots are similar with the y-values representing the features of importance with the top variable being the most important and the subsequent ones organized in descending rank of importance. Beeswarm plots must be plotted for a single target variable, in our case a student with a failure or a student without a failed course. In Figure 7, each dot represents a student record with the feature value represented by a color. As the feature value color bar shows, a higher value is pink and a lower value is blue. This helps us understand how the points are distributed and how the value of the variable may impact our model’s prediction. In the case of Figure 7, the most important feature on average is a student’s GRE scores, with students who passed all their courses tending to have lower GRE scores compared to students who failed a course (Figure 7B) tending to have higher GRE scores based upon the simulated data.

As we can see, the features of importance are different when using the SHAP approach. SHAP values did not rank age or pre-vet GPA as contributing to the model as greatly as the Gini criterion method did (Figure 6 and Figure 7 and Table 4). This may be explained by SHAP values not being as biased towards overestimating the importance of continuous variables, although it has been reported that in some datasets under certain conditions, SHAP has the potential to still be biased towards certain feature types such as numerical features with many unique values and categorical features with high cardinality [58]. Additionally, there is some discussion in the data science community about adding up the SHAP values for categorial variables that have been transformed with one-hot encoding to truly understand the categorical variable’s contribution to the model (e.g., https://towardsdatascience.com/shap-for-categorical-features-7c63e6a554ea (accessed on 1 May 2023)).

While our categorical data contained only five levels, one-hot encoding and dummy encoding may not be appropriate for handling categorical data with many different categorical levels (aka high-cardinality categorical variables). High cardinality categorical variables can lead to statistical problems [59], “dilute” the features of importance, and reduce the predictive performance of ML algorithms [59,60]. This means that other encoding methods such as hashing may need to be explored, or reducing or limiting the number of unique categories that are one-hot encoded.

Our mock dataset contained a low number of variables; however, often, academic datasets will contain a large number of variables. Feature selection, a way to reduce high dimensionality datasets, is routinely completed during the data preparation phase in order to reduce computation power, improve the performance of the ML algorithm, and to help with model interpretation [40]. It is important to consider the overall project goal as in veterinary education, it may be just as important to understand which variables are contributing only at a low level. Therefore, it may be best to consider developing models that undergo a feature selection step as well as a model which does not undergo feature selection.

It is important to recognize that this practical introduction to ML provides veterinary educators a basic knowledge of ML and to recognize how certain decisions, such as how to handle missing data, can impact ML models such as our example random forest model. While we cannot cover every aspect of building a ML model, there is one final consideration which is often overlooked, but has begun to be recognized in clinical datasets, and that is inclusion of under-represented populations. When demographic variables have categories that are highly underrepresented, this can result in bias [61,62] and provide potentially misleading conclusions. Consider our veterinary student population. Most colleges of veterinary medicine have a primarily white, female student population [63]; therefore, when incorporating demographic data, it is important to realize that low numbers of students from underrepresented minority groups, students who are or identify as male, and/or students who identify as LGTBQIA+ are some potential variables to consider may result in a biased model. Certain research questions, such as seeking to identify at-risk students, may need to be answered by incorporating a double prioritized bias correction technique which was recently published to correct bias in prognostic ML models where the dataset contained underrepresented racial and age groups [62]. In this approach to eliminating bias, custom ML models were built for each underrepresented group in addition to an all-encompassing ML model. This may be suitable for helping eliminate the under-representation bias that is present in the veterinary student population. Furthermore, this highlights one of many research priorities as ML begins to be incorporated into veterinary education.

Prediction models, such as the model described in this manuscript, are one of the primary uses of ML in higher education as the goal of predictive analytics is to identify students at-risk in mastering knowledge and successful course completion—enabling timely interventions [21,64]. Other potential uses of ML surrounding personalizing learning experiences for veterinary students include adaptive learning platforms. These platforms have been employed in medical education programs and use ML to analyze students’ performance and tailor the content based upon the identified strengths and weaknesses of the learner [65]. Furthermore, much interest exists in assessing veterinary skills and being able to provide objective, consistent, and immediate feedback to students. There is ongoing work to train AI on visual and physiologic data such as surface electromyographical (sEMG) data to use ML to help assess surgical skills of veterinary students [66].

## 5. Conclusions

Without established guidelines for handling data (i.e., MNAR) within the medical education field nor preferred methods for model evaluation, our results using simulated veterinary student data highlight the need for the veterinary educator to be fully transparent during the creation of ML models. We suggest future research efforts be directed toward establishing best practices within the education field for handling MNAR data and the other critical considerations.

## Figures and Tables

**Table 1:** All simulated variables for the student records are shown with the range of values or potential options. As an introductory primer for educators, some variables displayed in the table were limited to reduce the complexity of the analysis. The fail variable is the target variable with 0 describing a student who did not fail the veterinary school course (0 = no) and 1 describing a student who did fail the veterinary school course (1 = yes).

Variable Name	Range of Values	Type of Data
Full Name	400 randomly generated female and male names	Categorical
Gender	Male or Female	Categorical
Race/Ethnicity	Asian, Black, Latinx, Not Provided, White	Categorical
Age	20–40 years	Numeric
Pre-Vet School GPA	3.00–4.00	Numeric
GRE	260–330	Numeric
Fail	0–1	Numeric

**Table 2:** A confusion matrix defines the true negative (TN), false negative (FN), false positive (FP), and true positive (TP) values used in the calculation of the performance metrics. As shown in the chart, a TN outcome is when the model correctly predicts the student did not fail the course; a TP outcome is when the model correctly predicts the student failed a course; a FN outcome is when the model incorrectly predicts the student did not fail, but in reality the student did fail the course; and a FP outcome is when the model incorrectly predicts a student failed a course, but in reality the student did not fail the course.

	Actual Negative Class: 0, Student Who Did Not Fail	Actual Positive Class: 1, Student Who Did Fail
Predicted negative Class: 0, student who did not fail	True negative (TN)	False negative (FN)
Predicted positive Class: 1, student who did fail	False positive (FP)	True positive (TP)

**Table 3:** The performance metrics calculated when the random forest base model and the tuned random forest model was given the testing dataset.

Performance Metric	Random Forest Base Model	Radom Forest Tuned Model
Accuracy	87.07%	86.61%
Recall/Sensitivity/TPR	89.61%	89.77%
Specificity/TNR	87.15%	88.11%
Precision	86.46%	86.21%
F1-Score	86.24%	88.40%
ROC curve AUC	87.15%	88.11%

**Table 4:** The Gini criterion ranking of all features from each of the five random forest models created using the datasets. All GRE records dataset contained no missing values, whereas the missing low GRE values removed dataset removed any incomplete student records or were replaced by the mean values (missing low GRE values replaced with mean). The most important features for the randomly removed GRE scores with all student records eliminated that were incomplete is shown in the random missing GRE values removed columns and when those missing scores were replaced with the mean, the most important features are shown in the respective column.

All GRE Records	Feature Importance Score	Missing Low GRE Values Removed	Feature Importance Score	Missing Low GRE Values Replaced with Mean	Feature Importance Score	Random Missing GRE Values Removed	Feature Importance Score	Random Missing GRE Values Replaced with Mean	Feature Importance Score
GRE	0.241850	GRE	0.370290	GRE	0.357720	GRE	0.291419	preGPA	0.218575
Age	0.150457	Race_Not Provided	0.131981	preGPA	0.152793	preGPA	0.199777	GRE	0.181400
Race_Not Provided	0.146913	preGPA	0.106498	Age	0.146683	Age	0.163021	Age	0.180350
preGPA	0.130455	Age	0.100696	Race_Not Provided	0.096973	Race_Not Provided	0.071514	Race_Not Provided	0.117902
Race_White	0.078924	Race_White	0.093832	Race_Latinx	0.062529	Race_Asian	0.062693	Race_White	0.073569
Race_Latinx	0.067359	Gender_Male	0.082514	Race_White	0.057276	Race_White	0.059140	Race_Black	0.071190
Gender_Male	0.063287	Race_Latinx	0.047706	Race_Asian	0.044694	Race_Black	0.051953	Race_Asian	0.062513
Race_Black	0.063043	Race_Black	0.041844	Gender_Male	0.042020	Gender_Male	0.050608	Race_Latinx	0.060454
Race_Asian	0.057713	Race_Asian	0.024639	Race_Black	0.039311	Race_Latinx	0.049876	Gender_male	0.034048

## Data Availability

All simulated datasets and Python code is at https://github.com/RUSVMCenter4/Veterinary_Education_ML_Tutorial (accessed on 14 May 2023).
